# Enhancing Drug Efficacy against Mastitis Pathogens—An In Vitro Pilot Study in *Staphylococcus aureus* and *Staphylococcus epidermidis*

**DOI:** 10.3390/ani10112117

**Published:** 2020-11-15

**Authors:** Karthic Rajamanickam, Jian Yang, Saravana Babu Chidambaram, Meena Kishore Sakharkar

**Affiliations:** 1 College of Pharmacy and Nutrition, University of Saskatchewan, 107 Wiggins Road, Saskatoon, SK S7N 5E5, Canada; kar029@mail.usask.ca (K.R.); jian.yang@usask.ca (J.Y.); 2 Department of Pharmacology, JSS College of Pharmacy, JSS Academy of Higher Education & Research (JSS AHER), Mysuru-570015, Karnataka, India; saravanababu.c@jssuni.edu.in

**Keywords:** Staphylococcus, mastitis, combination therapy, drug targets, antibiotics

## Abstract

**Simple Summary:**

The success rate of antibiotic treatment of mastitis is highly variable. Concurrently, the efficacy of available antibiotics is compromised by the rapid emergence of drug-resistant bacteria. Recently, it was reported that there has been a reduction in the presence of antibiotic-resistant bacteria in food-producing animals where interventions provide for restrictions in antibiotic use. In addition, societal concerns regarding the use of antimicrobials in food animal production are putting increasing pressure on all aspects of livestock production. Here, we have conducted a systematic procedure for the identification of conserved and unique drug targets. We propose that combination therapy with drugs that work synergistically against conserved and unique targets can help increase efficacy and lower the usage of antibiotics for treating bacterial infections. An in vitro pilot validation of our findings in vitro for the two most common mastitis-causing bacteria in North America—*Staphylococcus aureus* and the coagulase-negative *Staphylococcus epidermidis*—is presented. We identified that the dosage of ceftiofur, the mostly used veterinary antibiotic, can be significantly reduced when used in combination with phytochemical phosphorylcholine.

**Abstract:**

Background: Bovine mastitis is one of the major infectious diseases in dairy cattle, resulting in large economic loss due to decreased milk production and increased production cost to the dairy industry. Antibiotics are commonly used to prevent/treat bovine mastitis infections. However, increased antibiotic resistance and consumers’ concern regarding antibiotic overuse make it prudent and urgent to develop novel therapeutic protocols for this disease. Materials and methods: Potential druggable targets were found in 20 mastitis-causing pathogens and conserved and unique targets were identified. Bacterial strains *Staphylococcus aureus* (ATCC 29213, and two clinical isolates CI 1 and CI 2) and *Staphylococcus epidermidis* (ATCC 12228, and two clinical isolates CI 1 and CI 2) were used in the present study for validation of an effective drug combination. Results: In the current study, we identified the common and the unique druggable targets for twenty mastitis-causing pathogens using an integrative approach. Furthermore, we showed that phosphorylcholine, a drug for a unique target gamma-hemolysin component B in *Staphylococcus aureus*, and ceftiofur, the mostly used veterinary antibiotic that is FDA approved for treating mastitis infections, exhibit a synergistic effect against *S. aureus* and a strong additive effect against *Staphylococcus epidermidis* in vitro. Conclusion: Based on the data generated in this study, we propose that combination therapy with drugs that work synergistically against conserved and unique targets can help increase efficacy and lower the usage of antibiotics for treating bacterial infections. However, these data need further validations in animal models of infection.

## 1. Introduction

Bovine mastitis results in large economic losses due to decreased milk production and increased production costs to the dairy industry. It compromises welfare for the affected cows and remains one of the most significant diseases affecting dairy cows worldwide. As the costliest disease in the Canadian dairy industry, culling rates due to mastitis are typically around 15% of dairy cows in a herd [1]. Mastitis has different levels of intensity and is caused by different organisms on and in cow udders. Exposure to microorganisms, host defense mechanisms, and environmental conditions are the three main factors involved in the etiopathology of bovine mastitis [2]. In dairy cows, more than 140 microorganisms have been reported to be involved in causing mastitis and several of these mastitis-causing pathogens infect beef cattle and bison as well. Moreover, the zoonotic potential of mastitis is high, due to the potential threat of bacteria and their toxins transferred by milk [3,4,5].

In North America, the antibiotics most widely used for the treatment of bovine mastitis are cephapirin, pirlimycin and ceftiofur [6]. Ceftiofur is a third-generation cephalosporin and is one of the most used antibiotics in dairy industry. Ceftiofur is labeled for veterinary use in the USA and Europe and is the drug of choice for the treatment of mastitis in the majority of dairy farms [7,8]. Ceftiofur inhibits bacterial cell wall synthesis. Oliver et al. evaluated the efficacy of extended ceftiofur intramammary therapy for treatment of subclinical mastitis in lactating dairy cows using the bacteriological cure rates based on negative culture 14 and 28 days after last treatment and reported that lengthening the duration of antibiotic therapy increased treatment efficacy in *Streptococcus uberis*, other environmental *Streptococcus* sp, and *Staphylococcus aureus* infections. They also reported that the cure rate for an 8-day extended ceftiofur treatment was 86% for coagulase-negative *Staphylococcus* sp, 80% for *Streptococcus dysgalactiae*, 70% for *Corynebacterium bovis*, 67% for *S. uberis, and* 36% for *S. aureus* [9].

However, overuse of antibiotics is a major problem in the treatment of bovine mastitis, and antibiotic treatment is frequently non-curative [10]. Moreover, with the growing demand for animal proteins, antibiotic residues in food and contamination of animal products with antibiotics has become a threat to public health. Hence, there is an urgent need to find novel therapeutic options and/or reduce the usage of antibiotics for treating bacterial infections in animals [11].

The availability of numerous bacterial genome sequences and the vast amount of biological information on bacteria provide an excellent resource for the identification of novel drug targets. One approach that can help fight the mounting threat of antibiotic resistance is the identification of novel antibiotic targets using genomic data of pathogenic bacteria. Towards this end, genomics can be applied to evaluate the “essentiality” and “selectivity” of the target. Earlier [12,13], we have shown that the target of interest has a greater chance of success as a lead if it is essential for the growth, replication, viability or survival of the microorganism, i.e., the target encodes for proteins/genes that are critical for pathogen’s survival in the host [14]. It has been observed that essential genes are important for basic biological processes in bacteria and hence have a greater likelihood to be conserved (common) across different genomes including the beneficial microbes in the human body. However, it must be noted that the development of essential genes as drug targets also increases the probability of the development of resistant strains and unintentional alterations to human health by subjecting beneficial microbes in the body to drugs and the consequent development of resistance [15]. One option is the identification of targets that are unique to the pathogen of interest. Interestingly, these unique targets (i.e., proteins that are present only in the pathogen of interest) may also help provide potential bacterial pathogenic-specific drug targets from given proteome(s) sequences [15].

Additionally, it is also important that the proposed bacterial target does not have a conserved homolog in the host, i.e., the target should be selective to the pathogen. This criterion helps address the cytotoxicity issues and can help avoid expensive dead-ends when a lead target is identified and investigated in great detail only to discover at a later stage that all of its inhibitors are invariably toxic to the host [13]. Furthermore, virulence factors assist the bacterium invade and colonize the host and are important for microbial pathogenesis. Virulence is the potential of an organism to infect the host and cause a disease. Virulent factors have been reported to facilitate evasion from the hosts’ immune defense mechanism, assistance in the acquiring of nutrients and dissemination of the bacteria within the host tissue [14,15]. The diverse range of virulence factors produced by the pathogens are important for the success of the pathogen as an infective agent [16]. Hence, virulent factors have been used for drug target prioritization and therapeutics in bacterial pathogens [17]. The predicted targets can then be explored to understand the pathophysiological genomics of the bacterium, and drugs that bind these targets can be explored as an arsenal against these bacteria [18].

Druggability can be predicted by the presence of protein folds (quaternary structures) that favor interactions with drug-like chemical compounds [19]. The binding of a small molecule to a protein with the appropriate binding affinity might make the protein druggable but does not necessarily make it a potential drug target. A protein of interest can be predicted to be druggable based on its sequence, structure or functional homology to a protein molecule that is confirmed to be druggable [20].

Various compounds such as phytochemicals and anti-metabolites have been reported to possess antibacterial action [21]. We have earlier shown that these two resources can be integrated to identify better treatments for bacterial infections including bovine mastitis. Our lab has also identified several antibiotic-phytochemical combinations for pathogenic bacteria [22]. Combination therapy can help broaden the antibacterial spectrum, treat polymicrobial infections, reduce the amount of antibacterial agents (if the two drugs are synergistic) and/or prevent the emergence of drug resistance. Here, we present a novel in silico (computational) approach that systematically identifies the potential common and unique targets for 20 mastitis-causing pathogens and the corresponding FDA-approved drugs against these targets.

The primary cause for the use of antibiotics in dairy farms is mastitis [23]. A broad-spectrum cephalosporin, ceftiofur, is active against Gram-positive and Gram-negative bacteria of veterinary importance [24,25,26]. It is currently approved for the treatment of mastitis infections and hence was chosen as the drug that is common as a therapeutic option in all the 20 bacteria. Phosphorylcholine was identified as a potential unique drug against gamma-hemolysin component B in *S. aureus* and *S. epidermidis* in our computational analyses.

*S. aureus* is a major mastitis-causing pathogen. It is highly contagious and has a significant impact on farm income [23]. One of the key concerns for livestock and public health and therapeutic failures is the emergence of methicillin-resistant *Staphylococcus aureus* (MRSA) [23,27]. Hence, there is a need to identify novel therapeutic options for treating *S. aureus* infections in dairy animals. *S. epidermidis* are coagulase negative *Staphylococcus* species (CNS). Mastitis infections caused by coagulase negative *Staphylococcus* species (CNS) are generally mild and usually remain sub-clinical [28,29]. However, CNS are the most common bovine mastitis isolates in many countries and are described as emerging mastitis pathogens that not only cause persistent infections and udder tissue damage but also lead to a somatic cell count (SCC) increase and a decrease in the quality of milk. Hence, we chose *S. epidermidis* as a representative strain of CNS and selected *S. aureus* and *S. epidermidis* for further analyses and validation in vitro. We validated the antimicrobial potential of a combination of ceftiofur and phosphorylcholine as a potential novel treatment for mastitis using these two bacteria.

We further investigated if drugs against common targets and unique targets when used in combination work synergistically.

We validated one combination of drugs (phosphorylcholine, a drug binding to a unique target in *S. aureus* and ceftiofur, a drug conventionally used to treat mastitis in dairy cattle). Our data demonstrate the in vitro efficacy of this combination in *S. aureus* and the coagulase-negative *S. epidermidis*, the two common mastitis-causing bacteria in North America.

## 2. Materials and Methods

### 2.1. Materials

Bacterial strains *Staphylococcus aureus* (ATCC 29213, and two clinical isolates CI 1 and CI 2) and *Staphylococcus epidermidis* (ATCC 12228, and two clinical isolates CI 1 and CI 2) were procured from Royal University Hospital, Saskatoon, Saskatchewan, Canada. Culture media (Brain-Heart Infusion broth (BHIB) and nutrient broth) and phosphorylcholine were purchased from Thermo Fisher Scientific (Ottawa, ON, Canada). Ceftiofur and all other chemicals used in this study were purchased from Sigma-Aldrich, Canada (Oakville, ON, Canada).

### 2.2. Methodology

#### 2.2.1. Prediction of “Essential” and “Specific” Targets in 20 Mastitis-Causing Bacteria

The proteomes of the host *Bos taurus* and the key mastitis-causing pathogens were downloaded from NCBI. *Bos taurus* proteome has 49,107 proteins. The number of proteins for each microorganism and genome identification IDs are listed in Table 1.

Essential genes are critical for survival and are largely determined by the organism’s environment. Gene essentiality data are commonly collected by mutagenesis in the selected gene of interest. Since the gene essentiality data were not available for the 20 mastitis-causing pathogens selected for analyses, we used the DEG (Database of Essential Genes) to compile a list of essential genes and their corresponding proteins in these pathogens [30]. Here, the proteomes of the 20 pathogens involved in mastitis were individually subjected to BLAST (Basic Local Alignment Search Tool) against the proteins in the DEG database (Database of Essential Genes) at an E-value cut-off of 10^−10^ and bit score >100 (Step-1). BLAST (Basic Local Alignment Search Tool) helps assign essentiality in silico based on homology. To minimize the issue of cross-reactivity of the drug due to the binding of the drug to homologous proteins in the host *Bos taurus* and exclude host proteins that are similar to the pathogen proteins, BLASTP analyses were also carried out for all the 20 mastitis-causing pathogens against *Bos taurus* proteome at an E-value cut-off of 10^−4^ and bit score >100. This approach helps select proteins that are essential for the 20 mastitis-causing pathogens and have no homologs in the host *Bos taurus* (Step-2). We further identified proteins that are essential (Step-1), not present in the host (Step-2) and are not annotated as hypothetical in the genome file (Step-3).

#### 2.2.2. Prediction of Druggable Targets and Drugs in 20 Mastitis-Causing Bacteria

Drugabbilty is the ability of a protein to be modulated by a drug like molecule. The druggability of the proteins encoded by essential genes was evaluated by screening against the DrugBank database https://www.drugbank.ca. The DrugBank database contains 8261 drug entries, including 2021 FDA-approved small molecule drugs, 233 FDA-approved biotech (protein/peptide) drugs, 94 nutraceuticals and over 6000 experimental drugs. Furthermore, 4338 non-redundant protein sequences (i.e., drug target/enzyme/transporter/carrier) are linked to these drug entries. We conducted a BLAST of the proteins (Step-3) against DrugBank. The resultant BLAST hits with bit score >100 and E-value cut-off of <10^−5^ were considered as potentially druggable therapeutic candidates (targets) (Step-4). We also identified common and unique drugs in DrugBank, for each of the 20 bacteria. These data are provided in Supplementary Tables S1 and S2.

#### 2.2.3. Prediction of Druggable Virulent Factors in 20 Mastitis-Causing Bacteria

The druggable proteins were further subjected to BLAST against VFDB (Virulence Factor Database) (Step-5). This helps identify proteins that code for virulent factors in the bacterium’s genome. The number of proteins predicted as potential virulent factors in the twenty mastitis-causing pathogens are listed in Table 1.

**Figure 1:**
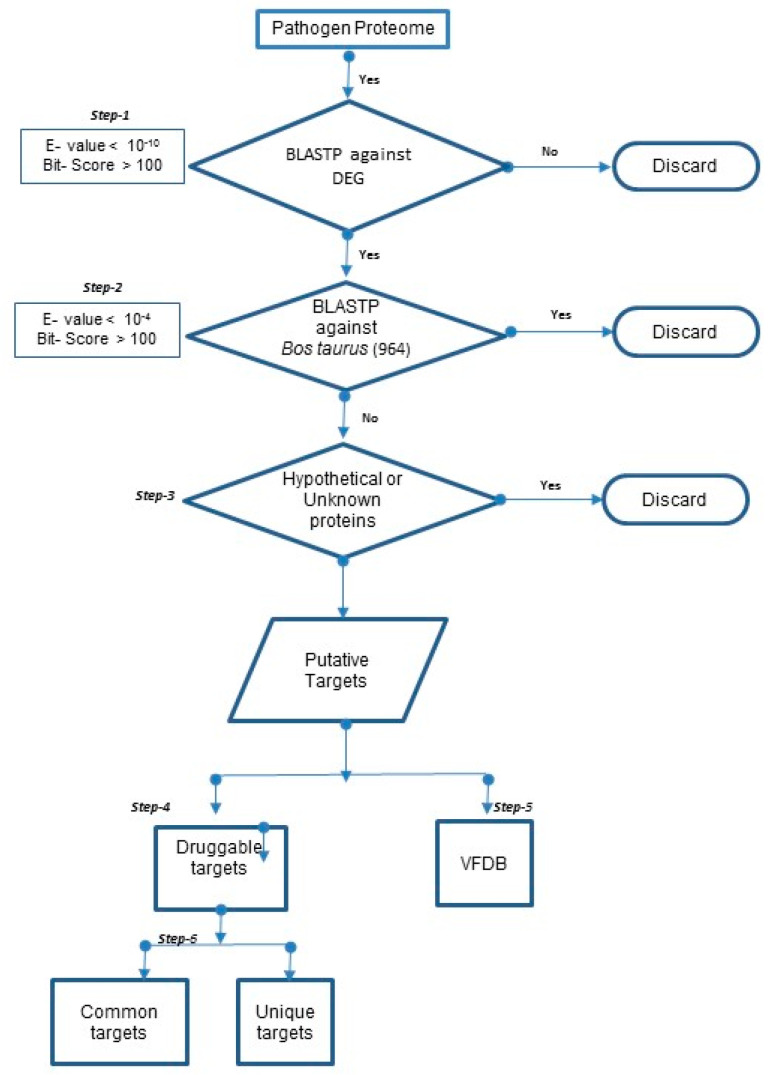
Flow chart for the process used for target prioritization in 20 mastitis-causing pathogens.

The flow chart for the process used for target prioritization is presented in Figure 1.

#### 2.2.4. Unique and Common Druggable Targets

We further identified conserved and unique drug targets by identifying resultant proteins that are present in all the 20 pathogens and resultant proteins that are specific to each bacterium, respectively (Step-6).

#### 2.2.5. Unique and Common Druggable Targets *Staphylococcus* Species

After computational analyses, we identified phosphorylcholine as a unique drug for *S. aureus* [28,29]. Phosphorylcholine has been reported to target gamma haemolysin protein component B (a virulent factor) in *S. aureus* (based on Drugbank database). Although we did not identify gamma haemolysin component B gene in *S. epidermidis* ATCC 12228 genome data, we identified the presence of haemolysin III, which is significantly homologous (based on sequence alignment) to gamma haemolysin at the protein level. Hence, we evaluated the efficacy of the combination of phosphorylcholine and ceftiofur (the FDA-approved drug used to treat mastitis) against these two pathogens [31].

#### 2.2.6. MIC for Phosphorylcholine and Ceftiofur and Their Combinations

Minimum inhibitory concentration (MIC) of the antimicrobial agents were determined by turbidity analyses. Standard broth micro dilution assay (CLSI) was used to determine the MICs of ceftiofur and phosphorylcholine for *S. aureus* and *S. epidermidis*. Briefly, the bacteria were sub-cultured in Nutrient Broth (NB) from −80 °C stock and subsequently incubated at 37 °C overnight. The bacterial suspensions were adjusted to 0.5 McFarland turbidity as per standard CLSI protocol (Approx. cell density 1.5 × 10^8^ CFU/mL) [32]. A total of 100 μL BHIB (Brain heart infusion broth) was added to each well of the 96-well plate followed by the addition of 5 μL/well of the bacterial suspension for all the bacterial strains under investigation.

For the determination of MICs, the phosphorylcholine concentration treatment ranged from 15.63 to 2000 μg/mL and the ceftiofur concentration treatment ranged from 0.02 to 1.25 μg/mL. The plates were incubated at 37 °C for 18–24 h and subsequently read at 595 nm using a 96-well plate reader (BIORAD iMark Microplate Reader, Mississauga, ON, Canada). Sensititre Vizion System (ThermoFisher Canada, Ottawa, ON, Canada) was also used to read the plates manually. Each experiment was performed in triplicate. The MIC of ceftiofur in combination with phosphorylcholine was evaluated using the checkerboard broth microdilution method. Here, two-fold serial dilutions of ceftiofur and phosphorylcholine were prepared. The phosphorylcholine concentration ranged from 15.63 to 2000 μg/mL and the ceftiofur concentration ranged was from 0.02 to 1.25 μg/mL The plates were incubated at 37 °C for 18–24 h and subsequently read at 595 nm.

Percentage of inhibition was calculated by using the formula (%) inhibition = {(OD of untreated control) − (OD of treated sample)/(OD of untreated control)} × 100. The fractional inhibitory concentration (FIC index) for the combinations was determined using the following formula. FIC index by checkerboard method was interpreted as follows: ≤0.5 is synergy; >0.5 and ≤4 is additive; and >4 is antagonism.

FIC of drug A = MIC of drug A when in combination/MIC of drug A when alone. Same for FIC of drug B.

## 3. Results

### 3.1. Prediction of Drug Targets in 20 Mastitis-Causing Bacteria

The bacteria that are reported in literature to be involved in causing mastitis are *Brucella melitensis*, *Corynebacterium bovis*, *Enterococcus faecalis*, *Enterococcus faecium*, *Escherichia coli*, *Klebsiella oxytoca*, *Klebsiella pneumoniae*, *Mycoplasma bovis*, *Nocardia abscessus*, *Pasteurella bettyae*, *Pasteurella dagmatis*, *Pasteurella multocida*, *Pseudomonas aeruginosa*, *Serratia liquefaciens*, *Staphylococcus aureus*, *Staphylococcus epidermidis*, *Streptococcus agalactiae*, *Streptococcus dysgalactiae*, *Streptococcus uberis*, and *Trueperella pyogenes*. This list is not exhaustive. The number of proteins in each bacterium are listed in Column C of Table 1.

The number of proteins that have a homolog in the DEG database and can be considered as potential drug targets (Step-1) is shown in Column D of Table 1.

The number of proteins that do not have a homolog in the *Bos taurus* proteome (Step-2), at the specified cut-off BLAST match score (as indicated in the methodology section), and can be considered as drug targets is shown in Column E of Table 1.

Proteins that were found to be essential (Step-1) and did not have a match in proteins in the *Bos taurus* genome (Step-2) and are not annotated as hypothetical can be considered as putative drug targets with less caution (Step-3) and are listed in Column G of Table 1.

It was also observed that several proteins had homology to virulent factors present in the VFDB database. The number of such proteins and the number of proteins that are putative targets and also have a homolog in the VFDB are listed in Column F and H of Table 1, respectively.

### 3.2. Prediction of Druggable Targets and Drugs in 20 Mastitis-Causing Bacteria

Out of the proteins identified in Step-3, the number of proteins that had a drug available in the drugbank database and can be considered as druggable targets (Step-4) (as indicated in the methodology section) is listed in Column I of Table 1 (Step-5).

The flow chart for the process used for target prioritization is presented in Figure 1.

### 3.3. Unique and Common Druggable Targets

There were 30 proteins identified as common druggable targets (Step-6). The number of conserved targets and their corresponding FDA-approved drugs for the 20 mastitis-causing pathogens are listed in Supplementary Table S1. As can be seen, most of the identified common druggable targets are ribosomal proteins and have an important function in translation.

### 3.4. Minimal Inhibitory Concentrations (MIC) and Fractional Inhibitory Concentrations (FIC)

**Figure 2:**
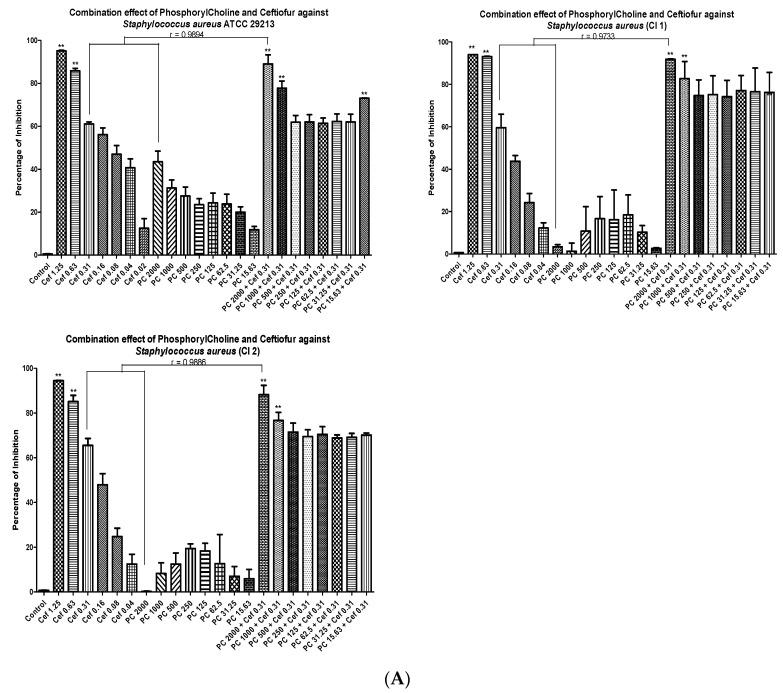

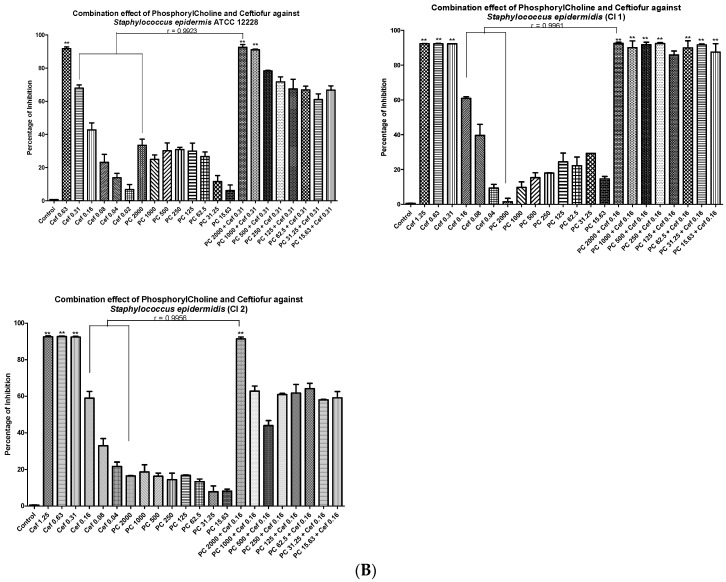
(**A**) Antimicrobial activity of ceftiofur (Cef), phosphorylcholine (PC) and their combination against *Staphylococcus aureus* (ATCC 29213, two clinical isolates CI 1 and CI 2). (**B**) Antimicrobial activity of ceftiofur (Cef), phosphorylcholine (PC) and their combination against *Staphylococcus epidermidis* (ATCC 12228, two clinical isolates CI 1 and CI 2). Pearson’s correlation was conducted between different experimental sets. Statistical analysis was performed using one-way ANOVA with ** representing *p*-value < 0.001 (n = 3).

The MICs of ceftiofur were found to be 0.63 for *S. aureus* (ATCC, CI 1 and CI 2) (Figure 2A) and 0.63, 0.31 and 0.31 µg/mL for the ATCC and the two clinical strains, C1 and C2 of *S. epidermidis* (Figure 2B), respectively. The MIC of phosphorylcholine was not achieved even at 2000 µg/mL for all the strains of *S. aureus* and *S. epidermidis* (Figure 2A,B).

The checkerboard broth microdilution assay was used to examine the synergistic/additive effect between phosphorylcholine and ceftiofur in ATCC and clinical strains of *S. aureus* and *S. epidermidis*. The antimicrobial activity was evaluated for ceftiofur at two sub-MIC doses. 0.31 μg/mL for *S. aureus* (ATCC, CI 1 and CI 2) and *S. epidermidis* (ATCC)) and 0.16 μg/mL for *S. epidermidis* (CI 1 and CI 2)) in combination with eight sub-MIC doses (2000, 1000, 500, 250, 125, 62.5, 31.25 and 15.63 μg/mL) of phosphorylcholine for all the strains. In total, 61% growth inhibition at a concentration of 0.31 μg/mL was observed for ceftiofur and 43.5% inhibition in growth was observed for phosphorylcholine at a concentration of 2000 μg/ML in *S. aureus* ATCC 29213, (Figure 2A). However, a significant increase in growth inhibition (86%) was seen as a result of the co-administration of 0.31 μg/mL ceftiofur and 2000 μg/mL phosphorylcholine (Figure 2A). An FIC index of 0.45 for the combination of ceftiofur and phosphorylcholine suggests strong synergy between ceftiofur and phosphorylcholine against *S. aureus*. For *S. aureus* clinical isolate 1 (CI 1), ceftiofur, a 59.57% inhibition in growth was observed at the concentration of 0.31 μg/mL, and 3.47% inhibition in growth was observed for phosphorylcholine at the concentration of 2000 μg/mL (Figure 2A). However, administration of 0.31 μg/mL ceftiofur and 2000 μg/mL phosphorylcholine in combination significantly increased the inhibition of growth to 91.82% (Figure 2A). The FIC index of the combination of ceftiofur and phosphorylcholine was calculated as 0.75 which suggests a strongly additive or weakly synergistic effect between ceftiofur and phosphorylcholine in *S. aureus* clinical isolate 1 (CI 1). For *S. aureus*, clinical isolate 2 (CI 2), ceftiofur showed a 65.52% inhibition in bacterial growth at a concentration of 0.31 μg/mL and phosphorylcholine showed a 0.29% inhibition in growth at a concentration of 2000 μg/mL (Figure 2A). However, 88.20% inhibition in bacterial growth was observed when 0.31 μg/mL ceftiofur and 2000 μg/mL phosphorylcholine were co-administered (Figure 2A). A strong additive or weak synergistic effect between ceftiofur and phosphorylcholine with an FIC index of 0.75 was observed in the case of *S. aureus* clinical isolate 2 (CI 2).

In total, 68% and 33% inhibition in growth was observed in *S. epidermidis* ATCC 12228 for Ceftiofur and phosphorylcholine at concentrations of 0.31 and 2000 μg/mL, respectively (Figure 2B). Simultaneous administration of 0.31 μg/mL ceftiofur and 2000 μg/mL phosphorylcholine increased the growth inhibition to 92.5% (Figure 2B). A strong additive or weak synergistic effect between ceftiofur and phosphorylcholine with an FIC index of 0.75 was observed for the combination of ceftiofur and phosphorylcholine against *S. epidermidis* ATCC 12228. For *S. epidermidis* clinical isolate 1 (CI 1), a 60.93% inhibition in growth was observed ceftiofur at a concentration of 0.16 μg/mL and phosphorylcholine showed 1.43% inhibition in growth at a concentration of 2000 μg/mL (Figure 2B). Co-administration of 0.16 ceftiofur and 2000 μg/mL phosphorylcholine increased growth inhibition to 92.49% (Figure 2B). The FIC index for ceftiofur and phosphorylcholine in combination was calculated to be 0.75, suggesting a strong additive or weak synergistic effect between ceftiofur and phosphorylcholine in *S. epidermidis* clinical isolate 1 (CI 1). For *S. epidermidis* clinical isolate 2 (CI 2), ceftiofur treatment shows 58.96% inhibition ingrowth at 0.16 μg/mL concentration and phosphorylcholine shows 16.51% inhibition in growth at a concentration of 2000 μg/mL (Figure 2B). Simultaneous administration of a combination of 0.16 μg/mL ceftiofur and 2000 μg/mL phosphorylcholine increased the growth inhibition to 91.42% (Figure 2B). A FIC index of 0.75 for the combination of ceftiofur and phosphorylcholine was calculated for *S. epidermidis* clinical isolate 2 (CI 2). This suggests a strong additive or weak synergistic effect between ceftiofur and phosphorylcholine in this bacterium was calculated against.

The FIC values for all the strains are shown in Table 2.

## 4. Discussion

The success rate of antibiotic treatment of mastitis is highly variable [10]. Concurrently, the efficacy of available antibiotics is compromised by the rapid emergence of drug-resistant bacteria [33]. Recently, it was reported that there is a reduction in the presence of antibiotic-resistant bacteria in food-producing animals where interventions provide for restrictions in antibiotic use [34]. Recent changes in regulations in Canada (December 2018) require a veterinary prescription for the use of medically important antimicrobials (antibiotics) (https://www.canada.ca/en/public-health/services/antibiotic-antimicrobial-resistance/animals/actions/responsible-use-antimicrobials.html) [35]. In addition, societal concerns regarding the use of antimicrobials in food animal production are putting increasing pressure on all aspects of livestock production. Consumers are becoming increasingly concerned about antibiotics in the food supply even though milk is rigorously tested for antibiotic residues [36]. Thus, there is an unmet need to discover and develop alternative treatments for mastitis that decrease dependency on antibiotics for treatment.

Conventionally, evolutionarily conserved proteins that are reported to be involved in essential functions have been explored as drug targets in pathogenic bacteria [37]. Essential proteins form the backbone for basic biological processes and play important roles for the lifestyle of the pathogen. Since these processes are shared in almost all bacteria, drugs against these conserved targets are generally highly non-specific and can cause side effects [37]. Moreover, it is imperative that the drugs should not cause cytotoxicity in the host. This can be achieved to some extent by targeting proteins that are present in the bacterium but are absent in the host, i.e., the criteria of selectivity. In this manuscript, we predicted targets that are essential and selective in 20 mastitis-causing bacteria and identified 30 potential conserved, selective and essential druggable targets in from bacteria. We also identified 104 FDA-approved drugs against these targets. Since these drugs have previously been approved, their redeployment as drugs against the mastitis-causing pathogens is an attractive approach because the toxicology and pharmacology profiles of these drugs are known. However, it is important to understand that bacterial genomes are diverse and have dynamic structures [38]. They have varying bioenergetic demands for adaptation that are involved in the regulation of the central metabolic pathways for survival [39]. Furthermore, bacterial proteins interact and form metabolic networks. Hence, it is crucial to understand redundancies and/or bypass mechanism/pathway within the metabolic networks formed by these druggable targets in each bacteria [39]. Nonetheless, these targets can provide some information on potential broad-spectrum drug targets in these pathogens.

On a parallel note, it is important to understand that antibacterial drugs targeting conserved proteins among bacteria have been reported as a causative factor for the development of drug resistance [15]. This is because these targets being involved in essential functions are also present in beneficial microbes which could become breeding grounds for the evolution of resistance or get destroyed by the drugs. Instead, unique genes can accelerate pathogen-specific drug target identification [15]. Drugs developed against unique genes have less chance of developing resistance and have less of an adverse impact on environment and friendly pathogens. This increases the chance of success as treatment therapeutics. Towards this concept, Chanumolu et al. designed a database to identify unique drug targets in pathogenic bacteria and explained its usage in *Mycobacterium tuberculosis* [15]. This server was, however, unavailable at the time of our current research. Unique genes, which are specific to the pathogen and absent from the host, essentially serve as potential drug targets as they not only avoid cross-reactivity and toxicity issues in the host genome but also ensure specific targeting of the organism of interest [15]. Here, we predicted unique drugs for the above 20 mastitis-causing pathogens. (Supplementary Table S2).

*S. aureus* and *coagulase-negative staphylococci* are two of the most frequently isolated mastitis pathogens in Canadian dairy farms [40]. Hence, we chose *S. aureus* and *S. epidermidis* for AST (antimicrobial susceptibility testing) of our predicted unique drugs in vitro. We identified phosphorylcholine as a drug against a unique target in the *S. aureus* genome. Phosphorylcholine is the precursor metabolite of choline in the glycine, serine and threonine metabolism pathways and also has a role in glycerophospholipid metabolism pathway. It forms pores in the membrane of *S. aureus* and causes toxicity (https://www.drugbank.ca/drugs/DB03945). Phosphorylcholine is also reported to bind the Gamma-hemolysin component B protein in *S. aureus*, though no reports were found on its action in *S. epidermidis.* Although implant-associated infection in orthopaedic defects was reported to be prevented by antibiotic-loaded phosphatidylcholine coatings [41], phosphorylcholine was not observed to be effective in our strains of *S. aureus* and *S. epidermidis.* This can be due to genomic heterogeneity among bacterial strains.

Since ceftiofur, an FDA-approved broad-spectrum third-generation cephalosporin, is the drug used for treating mastitis infections in cattle in Canada, we embarked on investing the AST of the combination of phosphatidylcholine with ceftiofur. Drug combinations are reported to have a better therapeutic efficacy compared to monotherapy against multi-drug-resistant bacterial pathogens [42] and can also delay the emergence of drug resistance [43]. Moreover, since synthetic antibiotics, even in combinations, have been reported to lead to the development of adaptive resistance over time [44,45], phosphatidylcholine being an antimetabolite provides a new option. Therefore, we evaluated its use in combinations with ceftiofur. We evaluated whether this combination is more effective in treating infections caused by *Staphylococcus* sp. and observed a synergistic effect of this combination in *S. aureus*. Interestingly, the drug combination also works additively/synergistically in *S. epidermidis*.

Here, it is important to mention that even though Gamma-hemolysin component B protein is present in *S. epidermidis*, it was not predicted as a target as being annotated as a hypothetical gene and hence it was not included for drugbank search. Furthermore, the concept presented above can be exploited not only to find pathogen-specific drug targets but also to study the diversity of a species, and provides an integrated knowledge-based approach for the development of novel drug combinations and next-generation targets for existing, withdrawn and inefficacious antimicrobials.

To date, there are no reports which have identified targets that are unique in *S. aureus*. This is the first report where FDA-approved drugs against common and unique targets in *S. aureus* have been shown to be synergistic. This approach can be easily expanded to other pathogens of interest. Moreover, with the decline in the development of new antibacterial drugs and the increase in the development of drug resistance among bacteria, combination therapy with drugs that work synergistically against conserved and unique targets can help increase efficacy.

Recently, Nobrega et al. reported that critically important antimicrobials (CIA) and non-CIA have comparable efficacy in treating non-severe bovine clinical mastitis caused by the most commonly reported bacteria that cause mastitis worldwide [46]. Moreover, it is important to mention that the use of third-generation cephalosporins may expose the dairy cattle to the risk of superinfection. Hence, caution (judicious use) is advised in its use alone or in combination therapy in veterinary medicine.

Limitations: Although BLAST homology search using computers makes it possible to hazard a “first-order guess” for the prediction of gene essentiality and druggability, experimental validations are essential for confirmation of the gene essentiality and druggability of a target before selecting a final list of targets for drug development. Moreover, it is important to confirm the prediction of unique cavity information for potential targets, so that the drugs designed against those cavities would not bind to beneficial/nonpathogenic organisms. This would help enhance the success of the proposed targets for further development.

Furthermore, it must be noted that phosphorylcholine is a major component of lipid membranes and already present in cows’ udders. Many bacteria us it to evade or take advantage of the immune system. Use of phosphorylcholine with subtherapeutic doses of ceftiofur may make the situation worse in a number of ways. However, it must be noted that combinations of ceftiofur and phosphorylcholine may help overcome resistant infections and resistance in case the first line and second line antibiotics are not effective in treating infections. Additionally, there is a possibility that the experimental dose concentration in vivo may work out to be higher than the normal concentration of phosphorylcholine inside cow udder and this may prevent the bacteria from taking advantage of the immune system. Further experiments are required to confirm the concentration of phosphorylcholine in vivo.

## 5. Conclusions

Reducing antibiotic usage and delaying/eliminating antibiotic resistance are important in treating bacterial infections specifically in dairy industry. In this study, we successfully applied an in silico approach to identify unique and common drug targets in 20 mastitis-causing bacteria. We propose that combination therapy with drugs working synergistically against conserved and unique targets can help increase efficacy and lower dosage of antibiotics for treating bacterial infections. Our findings were validated in vitro using two most common mastitis-causing bacteria in North America—*Staphylococcus aureus* and the coagulase-negative *Staphylococcus epidermidis.*

## Figures and Tables

**Table 1:** Summary of genomic analyses.

(A) Organism	(B) Genome ID	(C) # Proteins	(D) # Match DEG	(E) # Protein with No Match in *B. taurus*	(F) Proteins That Match in VFDB	(G) # of Putative Targets	(H) # of Putative Targets That Match in VFDB	(I) # of Druggable Targets
*B. melitensis*	NC_003317	2972	1380	2458	266	889	123	229
*C. bovis*	NZ_AENJ01000503	1829	850	1513	104	526	49	144
*E. faecalis*	NZ_KE351595.1	2732	1025	2380	221	902	166	339
*E. faecium*	NC_017960.1	3114	1076	2741	208	729	84	175
*E. coli*	NC_018658	5138	2240	4644	530	1646	262	491
*K. oxytoca*	NZ_CP011636	6816	2627	6293	589	2072	376	622
*K. pneumoniae*	NC_016845	5779	2397	5257	476	1743	279	533
*M. bovis*	NZ_CP007589	743	328	644	23	217	8	66
*N. abscessus*	NZ_BAFP01000274.1	7296	2054	6782	394	1482	229	564
*P. bettyae*	NZ_AJSX01000001	2059	1196	1756	160	852	90	226
*P. dagmatis*	NZ_GG704823	1980	1239	1651	193	865	105	237
*P. multocida*	NZ_CP008918	2013	1235	1700	192	885	107	236
*P. aeruginosa*	NC_002516	5572	2476	5105	765	2476	407	649
*S. liquefaciens*	NC_021741.1	4811	2246	4150	524	1722	142	521
*S. aureus*	NC_007795	2767	1143	2392	234	534	67	161
*S. epidermidis*	NC_004461	2482	1106	2114	164	636	73	173
*S. agalactiae*	NC_004116	2127	941	1827	186	602	69	149
*S. dysgalactiae*	NC_019042.1	1947	886	1641	157	586	53	145
*S. uberis*	NC_012004.1	1762	883	1451	139	592	51	145
*T. pyogenes*	NZ_JVLH01000002	1610	698	1328	92	455	38	119

DEG: Database of Essential Genes, VFDB: Virulence Factor Database. The genomes of 20 mastitis-causing organisms (A, B and C) were used to identify proteins that are essential for bacterial survival (D) and are absent in *B. taurus* (E). Bacterial proteins that are putative virulent factors were predicted (F). Number of proteins identified as putative targets (G) and the subset that is virulent factors (H) and is druggable (I) is listed.

**Table 2:** Fractional inhibitory concentration (FIC) values for the combinations been tabulated above.

No	Strain Name	MIC of Phosphoryl Choline (µg/mL)	MIC of Ceftiofur (µg/mL)	Synergistic Action of Phosphoryl Choline + Ceftiofur (µg/mL)	FIC Index Value	FIC Value Interpretation
1	*Staphylococcus aureus* ATCC 29213	>2000	1.25	2000 + 0.31	0.45	Synergistic
2	*Staphylococcus aureus* (CI 1)	>2000	0.63	2000 + 0.31	0.75	Strong additive or weak synergistic
3	*Staphylococcus aureus* (CI 2)	>2000	1.25	2000 + 0.31	0.75	Strong additive or weak synergistic
4	*Staphylococcus epidermidis* ATCC 12228	>2000	0.63	2000 + 0.31	0.75	Strong additive or weak synergistic
5	*Staphylococcus epidermidis* (CI 1)	>2000	0.31	2000 + 0.16	0.75	Strong additive or weak synergistic
6	*Staphylococcus epidermidis* (CI 2)	>2000	0.31	2000 + 0.16	0.75	Strong additive or weak synergistic

## Supplementary Materials

The following are available online at https://www.mdpi.com/2076-2615/10/11/2117/s1, Table S1: List of conserved essential targets in 20 mastitis-causing pathogens and their corresponding drugs from Drugbank database, Table S2: List of unique drugs from Drugbank database for each of the 20 mastitis causing pathogens.
