# Supplementary material for: Enhancing Drug Efficacy against Mastitis Pathogens—An In Vitro Pilot Study in Staphylococcus aureus and Staphylococcus epidermidis
Source: Animals (Basel). 2020 Nov 15;10(11):2117. doi: 10.3390/ani10112117 (PMC7696410; doi:10.3390/ani10112117)
Supplement: Supplementary file 1 [file animals-10-02117-s001.zip › animals-972573-SI.pdf]

**Supplementary Table S1: List of conserved essential targets in 20 mastitis-causing pathogens and their corresponding drugs from Drugbank database.**

| S.No | Count | Description | Drug |
|---|---|---|---|
| 1 | 1 | 30S ribosomal protein S10 | Nitrofurantoin |
| 2 | 14 | 30S ribosomal protein S12 | Framycetin; Amikacin; Tigecycline; Tobramycin; Gentamicin; Spectinomycin; Netilmicin; Neomycin; Streptomycin; Kanamycin; Ribostamycin; GENTAMICIN C1A; Arbekacin; 2-METHYLTHIO-N6-ISOPENTENYL-ADENOSINE-5'-MONOPHOSPHATE |
| 3 | 2 | 30S ribosomal protein S13 | Tigecycline; 2-METHYLTHIO-N6-ISOPENTENYL-ADENOSINE-5'-MONOPHOSPHATE |
| 4 | 3 | 30S ribosomal protein S19 | Tigecycline; Tetracycline; 2-METHYLTHIO-N6-ISOPENTENYL-ADENOSINE-5'-MONOPHOSPHATE |
| 5 | 2 | 30S ribosomal protein S3 | Tetracycline; 2-METHYLTHIO-N6-ISOPENTENYL-ADENOSINE-5'-MONOPHOSPHATE |
| 6 | 7 | 30S ribosomal protein S4 | Doxycycline; Lymecycline; Clomocycline; Oxytetracycline; Demeclocycline; Minocycline; 2-METHYLTHIO-N6-ISOPENTENYL-ADENOSINE-5'-MONOPHOSPHATE |
| 7 | 1 | 30S ribosomal protein S5 | 2-METHYLTHIO-N6-ISOPENTENYL-ADENOSINE-5'-MONOPHOSPHATE |
| 8 | 2 | 30S ribosomal protein S7 | Tetracycline; 2-METHYLTHIO-N6-ISOPENTENYL-ADENOSINE-5'-MONOPHOSPHATE |
| 9 | 2 | 30S ribosomal protein S8 | Tetracycline; 2-METHYLTHIO-N6-ISOPENTENYL-ADENOSINE-5'-MONOPHOSPHATE |
| 10 | 9 | 30S ribosomal protein S9 | Doxycycline; Lymecycline; Clomocycline; Tigecycline; Oxytetracycline; Demeclocycline; Minocycline; Rolitetracycline; 2-METHYLTHIO-N6-ISOPENTENYL-ADENOSINE-5'-MONOPHOSPHATE |
| 11 | 2 | 50S ribosomal protein L11 | 8-Hydroxy-4-(1-Hydroxyethyl)Quinoline-2-Carboxylic Acid; (4s)-2-[(1e)-1-Aminoprop-1-Enyl]-4,5-Dihydro-1,3-Thiazole-4-Carboxylic Acid |
| 12 | 1 | 50S ribosomal protein L16 | Chloramphenicol |
| 13 | 5 | aminotransferase class V | Chloramphenicol; Selenocysteine; S-Mercaptocysteine; S-Selanyl Cysteine; L-2-amino-3-butynoic acid; 3'-O-N-Octanoyl-a-D-Glucopyranosyl-B-D-Fructofuranoside |
| 14 | 19 | DNA gyrase subunit A | Ciprofloxacin; Rosoxacin; Moxifloxacin; Grepafloxacin; Enoxacin; Pefloxacin; Trovafloxacin; Cinoxacin; Lomefloxacin; Norfloxacin; Levofloxacin; Gemifloxacin; Ofloxacin; Sparfloxacin; Temafloxacin; Fleroxacin; Besifloxacin; Finafloxacin; Gatifloxacin |
| 15 | 5 | DNA ligase (NAD(+)) LigA | N-[2-(2,4-diaminopyrido[2,3-d]pyrimidin-7-yl)-2-methylpropyl]-4-phenoxybenzamide; Nicotinamide Mononucleotide; 2-amino-7-fluoro-5-oxo-5H-chromeno[2,3-b]pyridine-3-carboxamide; 7-amino-2-tert- |

| | | | |
|---|---|---|---|
| | | | butyl-4-{[2-(1H-imidazol-4-yl)ethyl]amino}pyrido[2,3-d]pyrimidine-6-carboxamide; 7-amino-2-tert-butyl-4-(4-pyrimidin-2-ylpiperazin-1-yl)pyrido[2,3-d]pyrimidine-6-carboxamide |
| 16 | 4 | DNA recombination/repair protein RecA | Phosphoaminophosphonic Acid-Adenylate Ester; Adenosine-5'-Diphosphate Monothiophosphate; 2'-Deoxyadenosine 5'-Triphosphate; Tetrafluoroaluminate Ion |
| 17 | 5 | DNA topoisomerase I | Thymidine-5'-Phosphate; Adenosine-3'-5'-Diphosphate; Thymidine-3',5'-Diphosphate; Pefloxacin; Novobiocin |
| 18 | 3 | DNA-directed RNA polymerase subunit alpha | Rifabutin; methyl [(1E,5R)-5-{(3S)-3-[(2E,4E)-2,5-dimethylocta-2,4-dienoyl]-2,4-dioxo-3,4-dihydro-2H-pyran-6-yl}hexylidene]carbamate; Myxopyronin B |
| 19 | 1 | guanylate kinase | Formic Acid |
| 20 | 2 | Holliday junction DNA helicase RuvB | Hexane-1,6-Diol; Adenine |
| 21 | 1 | methionyl-tRNA formyltransferase | N-Formylmethionine |
| 22 | 1 | phenylalanine--tRNA ligase subunit beta | 1-{3-[(4-pyridin-2-ylpiperazin-1-yl)sulfonyl]phenyl}-3-(1,3-thiazol-2-yl)urea |
| 23 | 1 | phosphate acetyltransferase | Acetylphosphate |
| 24 | 1 | ribosome biogenesis GTPase Der | Guanosine-5'-Diphosphate |
| 25 | 1 | ribosome maturation factor | Decyloxy-Methanol |
| 26 | 1 | RNA methyltransferase | S-Adenosyl-L-Homocysteine |
| 27 | 3 | RNA polymerase sigma factor | Fidaxomicin; Myxopyronin B; methyl [(1E,5R)-5-{(3S)-3-[(2E,4E)-2,5-dimethylocta-2,4-dienoyl]-2,4-dioxo-3,4-dihydro-2H-pyran-6-yl}hexylidene]carbamate |
| 28 | 2 | thioredoxin reductase | Flavin adenine dinucleotide; Azelaic Acid |
| 29 | 1 | tRNA (guanosine(37)-N1)-methyltransferase TrmD | S-Adenosyl-L-Homocysteine |
| 30 | 1 | uracil phosphoribosyltransferase | Uridine-5'-Monophosphate |

**Supplementary Table S2: List of unique drugs from Drugbank database for each of the 20 mastitis causing pathogens.**

Please see the attached .xls file for drugs.
